# Genetic diversity and population structure of indigenous guinea fowl (*Numida meleagris)* in Benin using simple sequence repeat markers

**DOI:** 10.5194/aab-66-183-2023

**Published:** 2023-07-14

**Authors:** Boko Michel Orounladji, Venant Houndonougbo, Mahugnon Santoze Adido, Aïcha Edith Soara, Blaise Arnaud Hako Touko, Christophe A. A. M. Chrysostome, Koffi Tozo

**Affiliations:** 1 Centre d'Excellence Régional sur les Sciences Aviaires, Université de Lomé, Lomé, Togo; 2 Faculté des Sciences Agronomiques, Université d'Abomey-Calavi, Abomey-Calavi, Benin; 3 Department of Animal, Aquaculture and Range Sciences, Sokoine University of Agriculture, Morogoro, Tanzania; 4 Département Environnement et Forêts, Institut de l'Environnement et de Recherches Agricoles, Centre National de la Recherche Scientifique et Technologique, Ouagadougou, Burkina Faso; 5 Biotechnology and Bioinformatics Research Unit, Department of Animal Science, University of Dschang, Dschang, Cameroon; 6 Faculté des Sciences, Université de Lomé, Lomé, Togo

## Abstract

This study aimed to assess the genetic diversity and population structure of 12 guinea fowl phenotypes from three climatic zones (Guinean, Sudano-Guinean and Sudanian) of Benin. A total of 96 adult guinea fowl, aged at least 6 months, were selected for blood sampling. Fragment analysis was carried out using 17 polymorphic microsatellite markers. The informative marker combinations revealed a total of 83 alleles across all loci, with an average of 5 alleles per locus and a mean polymorphic information content (PIC) of 0.793. This study showed an observed heterozygosity of 0.492. The inbreeding coefficient values ranged from - 0.086 in white phenotype to 0.226 in cinnamon, showing a deficit of heterozygotes, suggesting a moderate inbreeding level. A relatively low population differentiation was observed, with a mean fixation index (Fst) value of 0.033. The short genetic distances between phenotypes, unlike the strong genetic identities, revealed high genetic proximity between the 12 phenotypes of indigenous guinea fowl in Benin. These data indicate the existence of a single indigenous guinea fowl population with high intra-population genetic diversity with respect to climatic zones or phenotypes. This study will help in the selection of parental breeding stock for genetic improvement programs, as well as in the conservation for biodiversity maintenance and sustainable use of the indigenous guinea fowl in the study zones in Benin.

## 1 Introduction

Livestock is a key driver of sustainable agricultural development. It contributes to food security, nutrition, poverty reduction and economic growth (FAO, 2020). Poultry farming, which is an integral part of the livestock subsector, is an important economic activity in many African countries. It is characterized by rich and diversified poultry genetic resources. Indigenous guinea fowl phenotypes contribute to rural livelihoods despite their low productivity. Animal genetic resources make a large contribution to food production, but more and more, these resources are threatened by the loss of their genetic identity (Adeola et al., 2015). Thus, local genetic resources have a great role to play in the context of climate change and where the criterion of adaptation, an attribute of local resources, is a great difficulty for exotic breeds in tropical environments (Naves et al., 2011). There is evidence that new breeding practices contribute to the modification of the diversity of animal genetic resources through the creation of new breeds and the improvement of lower-performing breeds (FAO, 2007). Among these resources is guinea fowl, which contributes to the livelihoods of rural populations. Indigenous guinea fowl ecotypes represent a reservoir of useful guinea fowl genomes and genes, which are desirable for their adaptability to production under a wide range of environmental conditions. Due to the harsh environmental conditions in which indigenous guinea fowl are raised, they may contain genes and alleles important for their adaptation to particular environments (Osei-Amponsah et al., 2010). Despite all the advantages of this indigenous resource, its productivity is low and little is known about its genetic diversity. Indeed, their reproductive performance can be improved by raising them in appropriate habitats under strict sanitary control and subjecting them to a balanced diet (Orounladji et al., 2022). However, considering only these environmental factors (reproduction, feeding, health) would omit the genotypic factor, which is also very crucial in a genetic improvement program. The study of the genetic diversity of indigenous populations does indeed appear to be interesting to distinguish the genes which have economic importance for poultry farmers (Fathi et al., 2017). According to several authors (Oguntunji, 2013; Haoua et al., 2015; Ould Ahmed and N'Daw, 2015), the characterization and conservation of various genetic attributes of indigenous species are imperative and long overdue in order to maintain the genetic biodiversity of these animals, improve food security of the rural households and enhance economic empowerment in developing countries. Thus, there is a need for a preservation policy for this important local breed. In order to effectively conserve and improve animal genetic resources, it is essential to provide a thorough and comprehensive description of the genetic diversity present within the current populations (FAO, 2013). The characterization of animal genetic resources provides appropriate data for their conservation and sustainable management for food security and economic development (Osei-Amponsah et al., 2017). Information on genetic diversity is essential for decision-making in the implementation of animal genetic resources improvement programs (FAO, 2013).

To this end, molecular markers offer the great advantage of allowing a direct assessment of genetic diversity, which is by definition independent of environmental effects (Ollivier et al., 2000). Molecular markers, including simple sequence repeats (SSRs) or microsatellites, have become favored markers in population genetics due to their genome-wide dispersion, high polymorphism and other advantageous characteristics (Atallah and Subbarao, 2011; Mtileni et al., 2012; Mahammi, 2015).

According to some authors, markers developed or identified for one avian species can be used in others in the same taxonomic family (Kayang et al., 2002, 2010; Botchway et al., 2013; Weimann et al., 2016; Traoré et al., 2018). Studies have highlighted that polymorphisms of microsatellite marker loci developed in chicken and quail have been revealed in guinea fowl (Kayang et al., 2002; Botchway et al., 2013; Weimann et al., 2016). An awareness of African states in the south of the Sahara has prompted numerous studies in recent years on the genetic diversity of indigenous animal resources, including indigenous guinea fowl populations. These studies have focused on the genetic diversity and phylogenetic structure of these locally available guinea fowl in Ghana (Kayang et al., 2010; Botchway et al., 2013), the Republic of the Sudan (Weimann et al., 2016), Burkina Faso (Traoré et al., 2018) and Togo (Soara et al., 2022).

However, information on the genetic characterization of indigenous guinea fowl in Benin is almost non-existent. Except for the summary phenotypic characterization conducted by Chrysostome (1995) and Houndonougbo et al. (2017), only the recent study conducted on morphobiometric characterization and biodiversity has provided information on morphological and morphometric diversity within the indigenous guinea fowl population distributed in the three climatic zones of Benin (Orounladji et al., 2021). Regarding molecular genetic diversity, no study to the best of our knowledge has been conducted so far on the indigenous guinea fowl resources of Benin taking into account all phenotypes identified in all climatic zones. To contribute to filling this gap, this molecular characterization was undertaken in the three climatic zones of Benin in order to determine the molecular genetic diversity and the structure of these local guinea fowl populations.

**Figure 1:**
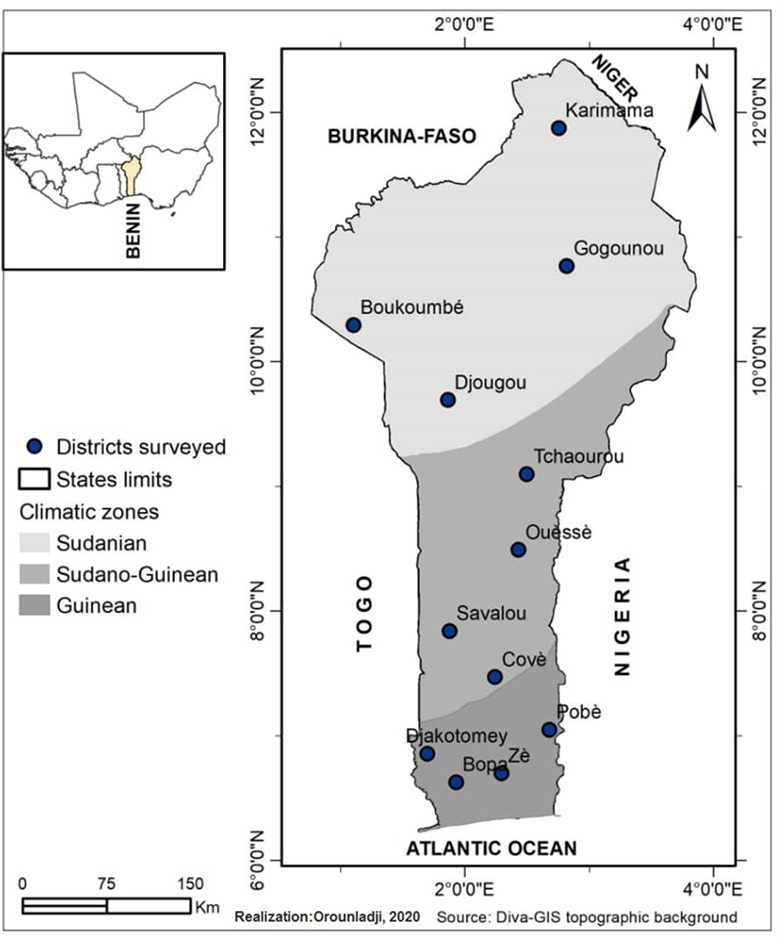
Map of climatic zones in Benin with the locations of guinea fowl sampled (Orounladji et al., 2021).

## 2 Materials and methods

### 2.1 Study areas and sampling

**Figure 2:**
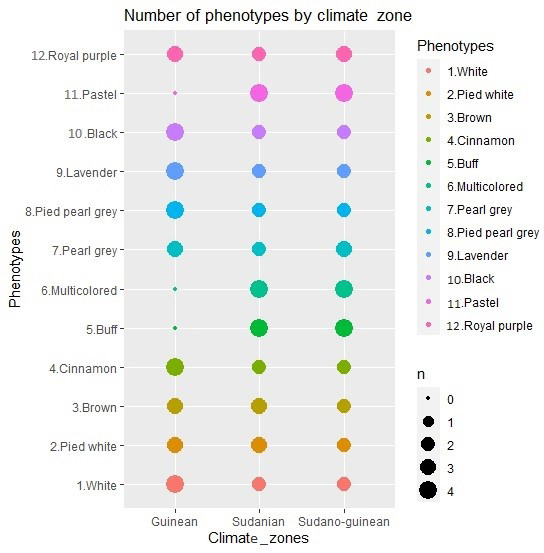
Number of phenotypes sampled by climate zone.

The study was carried out in the 12 districts of the 3 climatic zones of Benin: the Sudanian zone (9 ${}^{\circ }$ 45 ${}^{\prime }$ –12 ${}^{\circ }$ 25 ${}^{\prime }$ N), the Sudano-Guinean zone (7 ${}^{\circ }$ 30 ${}^{\prime }$ –9 ${}^{\circ }$ 45 ${}^{\prime }$ N) and the Guinean zone (6 ${}^{\circ }$ 25 ${}^{\prime }$ –7 ${}^{\circ }$ 30 ${}^{\prime }$ N) (Fig. 1). A total of 96 adult guinea fowls of at least 6 months of age were selected for blood sampling. Thus, 32 samples were collected from each climatic zone on the basis of plumage color. In total, 8 samples for each of the 12 identified phenotypes were analyzed. The phenotypic representation of the samples by climatic zone is detailed in Fig. 2. The morphobiometric characteristics of all phenotypes are contained in our previous studies (Orounladji et al., 2021). In order to select unrelated individuals, the farmers were not only asked to provide the match between the sampled individuals, but they were also asked to select no more than two individuals per farm.

### 2.2 Blood sample collection

For each sample, a volume of 1.5 mL of blood was drawn into vacutainer tubes containing ethylenediaminetetraacetic acid (EDTA) from the wing vein with single-use 2.5 mL syringes fitted with 22G × 1 1/4 needles. After a swift stirring of the contents of the tubes (blood + anticoagulant) without strong agitation, the samples were kept in a freezer at - 20 ${}^{\circ }C$ before the DNA extraction.

### 2.3 DNA extraction

Genomic DNA was extracted from whole blood using the Quick-DNA™ Miniprep Plus Kit (Zymo Research, catalogue no. D4068) according to the manufacturer's recommendations. DNA samples were stored at 4 ${}^{\circ }C$ until gene amplification (PCR) and genotyping.

**Table 1:** Characteristics of the SSR markers.

Multiplexes	Loci	Allele size ( bp )	Fluorochrome	Accession number	Primers (5 ${}^{\prime }$ -3 ${}^{\prime }$ )
Multiplex 1	GF12	100–110	FAM	G4QAAT301BPAU2	F: GCACTAATAGTAGAGTACGCAGAAR: TGCTAACTCCAAATGACACA
	GUJ0013	134–150	FAM	AB035823	F: ACCAAACCCGAGATCCGACAR: AGCGTTCGCGTTCCTCTTTC
	GF168	214–230	FAM	G4QAAT301BZOWA	F: GGCCCTATCCTCAAATAGTCTCCR: TCAAAGCCTGTAAGAAGTGCTC
	GF43	111–117	ATTO532	G4QAAT301BEYNH	F: TCTGAAGTATCTGCCCTGAGR: TTATCAAGTGAGCGATCAGA
	GF69	182–200	ATTO532	G4QAAT301BRYSU	F: TTCCAGCTTGAAAACTGACTR: CACAGACACAGACCATTGAG
	GUJ0084	165–167	ATTO550	AB063152	F: ACTCCTCCTCTTTCTCCCTCR: TCCCGTCTCCCGATGTGTTT
	GUJ0059	211–231	ATTO550	AB063127	F: GACAAAGTTACAGCTAGGAGR: TAGGTGCGAAAATCTCTGAC
Multiplex 2	GF13	114–136	FAM	G4QAAT301BX99P	F: TGTACATGGTGCGTGTTTATR: CGTTTTTGTCCGTACTCAAC
	GF74	208–214	FAM	G4QAAT301ACCH6	F: AACCTGCAGAAACACATTTTR: CTGCAATACTTCATTTGTGG
	GF5	165–167	ATTO532	G4QAAT301BHEM5	F: GTCTTCTCTGACTTTTGGAAATR: TACCCACACTGGTACTCTCC
	GUJ0001	222–226	ATTO532	AB035652	F: GAAGCGAAAGCCGAGCCAR: CAGCACTTCGGAGCACAGGA
	GF30	192–202	ATTO550	G4QAAT301B1L8O	F: AACAAAGGATGTTTTGTGCTR: TAAACCAATTTCCAGCATTT
	GF75	207–217	ATTO565	G4QAAT301A76GJ	F: TCTCTCCTGACTTCCAAAAAR: AGGCTTGAACTCATGGACTA
Multiplex 3	MCW0069	182–200	FAM		F: GCACTCGAGAAAACTTCCTGCGR: ATTGCTTCAGCAAGCATGGGAGGA
	GF37	206–230	ATTO532	G4QAAT301BB71Y	F: TCTTCCTTCAGAGGTACCAAR: TGAAGACCATAGAAGCCTGT
	GUJ0086	205–213	ATTO550	AB063154	F: AGCTGCCATATCTACTGCTCR: TGGCTTAGTGCTTTCAGAGG
	GUJ0066	161–255	ATTO565	AB063134	F: GGGAAAACAATCACTGCCTCR: TCTGCAAATCCCCCTTAGAG

F: forward primer; R: reverse primer

### 2.4 Amplification of microsatellite markers and genotyping

Polymerase chain reactions (PCRs) were performed on gDNA from each guinea fowl, using specific primers for each microsatellite marker. A set of 17 microsatellite markers were chosen for this study: 10 microsatellite markers (GF5, GF12, GF13, GF30, GF37, GF43, GF69, GF74, GF75 and GF168) developed by Botchway et al. (2013) for guinea fowl; 6 microsatellite markers (GUJ0001, GUJ0013, GUJ0059, GUJ0066, GUJ0084 and GUJ0086) developed by Kayang et al. (2002) for guinea fowl, quails and hens; and 1 microsatellite marker (MCW0069) from the panel recommended by the FAO for the study of diversity in hens. Each of them with forward primer was conjugated to one of the four fluorescent dyes FAM (blue), ATTO532 (green), ATTO550 (yellow) and ATTO565 (red). These markers were chosen based on their technical characteristics (good aptitude for amplification and easy interpretation of typing) and their genetic characteristics (number of alleles, localization and coverage of the genome). The characteristics of these microsatellite markers are presented in Table 1.

The amplifications were carried out in a thermal cycler using the following conditions: initial denaturation at 94 ${}^{\circ }C$ for 3 min , followed by 30 cycles of 30 s at 94 ${}^{\circ }C$ , 60 s at annealing temperature ranging from 45 to 68 ${}^{\circ }C$ (which is determined by the temperature of each primer pair) and 60 s extension at 68 ${}^{\circ }C$ , and then final extension at 68 ${}^{\circ }C$ for 5 min ended the reactions.

### 2.5 PCR reactions for tagged simplex reactions

The PCR was conducted in three multiplexes (Table 1), and each multiplex was formed according to specific criteria. For the alleles having almost the same fragment size, different fluorochrome dyes were considered for the grouping, whereas different markers having a similar fluorochrome dye and almost a similar fragment size were assigned to a different multiplex group.

The microsatellite regions were first amplified using One*Taq* ^®^ 2X Master Mix with Standard Buffer (NEB), using a PCR reaction volume of 20 µL containing at least 10 ng of gDNA , 10 mM of each individual tagged primer, and 10 µL One*Taq* ^®^ 2X Master Mix and nuclease-free water.

The second round of PCR was conducted in the same way using 1 µL of Round-1 PCR product as a template and the fluorescently labeled M13-FAM, PGEX3-ATTO532, PGEX5-ATTO550 and T7-ATTO565 forward primers and corresponding reverse primers.

### 2.6 PCR reactions for tagged multiplex reactions

Similar to above, the microsatellite regions were first amplified using Q5 High-Fidelity 2X Master Mix catalogue no. M0292 (NEB), using a PCR reaction volume of 20 µL containing at least 10 ng of gDNA , 10 mM of each primer in a primer mix, and 10 µL Q5 High-Fidelity 2X Master Mix and nuclease-free water. This round of PCR adds the M13, PGEX3, PGEX5 and T7 sequences to the PCR product.

The second round of PCR was conducted in a similar fashion as above, using 1 µL of Round-1 PCR product as a template and the fluorescently labeled M13-FAM, PGEX3-ATTO532, PGEX5-ATTO550 and T7-ATTO565 forward primers and corresponding reverse primers in a primer mix.

### 2.7 Preparation of fragments

The fluorescently labeled PCR amplicons were diluted at a ratio of 1 : 10. The diluted amplicons were then mixed with the LIZ500 sizing standard and highly deionized formamide (catalog no. 4311320, Thermo Fisher Scientific, Carlsbad, USA). This mixture was denatured at 95 ${}^{\circ }C$ for 5 min. After denaturation, the fragments were run on the ABI PRISM™ 3500xl Genetic Analyzer (Applied Biosystems, Thermo Fisher Scientific, Carlsbad, USA), 50 cm capillary array, POP-7™. The data were analyzed and interpreted using GeneMarker V2.9.5 (SoftGenetics, State College, Pennsylvania, USA).

**Table 2:** Heterozygosity, Wright's statistical F and polymorphism in Guinean, Sudano-Guinean and Sudanian populations of guinea fowl in Benin.

Loci	$N_{a}$	$N_{e}$	I	$H_{o}$	$H_{e}$	PIC	$F_{\text{IS}}$	$F_{\text{ST}}$	$F_{\text{IT}}$
GF12	6	2.947	1.231	0.646	0.646	0.696	- 0.001	0.045	0.044
GUJ0013	8	3.129	1.299	0.781	0.674	0.637	- 0.160	0.017	- 0.141
GF168	7	3.438	1.358	0.677	0.707	0.734	0.043	0.011	0.053
GF43	5	2.456	0.999	0.385	0.588	0.858	0.344	0.051	0.378
GF69	6	2.763	1.259	0.313	0.625	0.696	0.500	0.037	0.519
GUJ0084	2	1.879	0.655	0.344	0.463	0.958	0.258	0.057	0.300
GUJ0059	6	3.918	1.437	0.521	0.745	0.702	0.301	0.021	0.315
GF13	7	2.611	1.177	0.469	0.594	0.696	0.211	0.053	0.253
GF74	6	2.847	1.284	0.646	0.630	0.626	- 0.026	0.020	- 0.005
GF5	3	2.453	0.986	0.438	0.589	0.907	0.258	0.018	0.271
GUJ0001	3	1.137	0.269	0.125	0.118	0.907	- 0.059	0.009	- 0.050
GF30	4	2.875	1.187	0.542	0.642	0.834	0.157	0.083	0.226
GF75	3	2.443	0.954	0.594	0.588	0.907	- 0.011	0.018	0.007
MCW0069	4	2.709	1.097	0.469	0.629	0.858	0.255	0.056	0.297
GF37	5	3.329	1.347	0.573	0.699	0.740	0.180	0.023	0.199
GUJ0086	3	2.578	1.013	0.479	0.606	0.907	0.210	0.023	0.228
GUJ0066	5	2.390	1.015	0.363	0.578	0.827	0.372	0.018	0.384
Mean	5	2.700	1.092	0.492	0.595	0.793	0.167	0.033	0.193
Standard error	0.190	0.100	0.043	0.025	0.020	0.108	0.043	0.005	0.043

$N_{a}$ : number of alleles, $N_{e}$ : number of effective alleles, I : Shannon's information index, $H_{o}$ : observed heterozygosity, $H_{e}$ : expected heterozygosity, PIC: polymorphism information content, $F_{\text{IS}}$ : inbreeding coefficient; $F_{\text{ST}}$ : fixation index; $F_{\text{IT}}$ : overall fixation index.

### 2.8 Data analysis

The frequency of null alleles was checked first in the dataset using Micro-Checker 2.2.3 (Van Oosterhout et al., 2004). This step was followed by an adjustment of the allele and genotype frequencies of the amplified alleles, for their use in further population genetic analysis.

Observed heterozygosity ( $H_{o}$ ) and expected heterozygosity ( $H_{e}$ ) (Nei, 1978), considered as basic genetic diversity measures, were calculated using GenAlEx 6.5 software (Peakall and Smouse, 2012). Pairwise and global F statistics were estimated using Genepop 4.2.2 software (Rousset, 2008). The estimation of genetic differentiation between populations was made using the $F_{\text{ST}}$ coefficient (Weir and Cockerham, 1984). The Hardy–Weinberg equilibrium (HWE) for each locus within populations was estimated by $F_{\text{IS}}$ statistics (Weir and Cockerham 1984), using the exact test included in Genepop 4.2.2 (Rousset, 2008).

Pairwise genetic distances (Nei, 1972) between subpopulations were estimated using GenAlEx 6.5 software (Peakall and Smouse, 2012). To investigate relationships between subpopulations, neighbor-joining (Saitou and Nei, 1987) dendrograms were constructed from Nei's genetic distances using the DendroUPGMA online platform (Garcia-Vallve et al., 1999). The factorial neighbor-joining trees based on inter-individual allele-sharing distances among the 12 phenotypes and 3 climatic zones were drawn using DARwin 6.0.021 software (Perrier and Jacquemoud-Collet, 2019). Phenotype differentiation based on genetic information was performed using the test proposed by Raymond and Rousset (1995). After defining groups of phenotypes based on their historical origin, a hierarchical analysis of the variance was carried out using the analysis of molecular variance. The factorial component analysis was performed using DARwin 6.0.021 software (Perrier and Jacquemoud-Collet, 2019) to visualize the differences between the studied subpopulations of indigenous guinea fowl.

The genetic population structure analysis of the 12 guinea fowl subpopulation was assessed using the Bayesian admixture procedure implemented in STRUCTURE 2.3.4 to infer the most likely number of clusters (Pritchard et al., 2000). The software had been programmed to run using the admixture model and correlated allele frequencies. The estimation of the number of assumed populations ( K ) was done for K ranging from 2–12. Five replicates were routed per each value of K with a burn-in period of 100 000 followed by 100 000 Markov chain Monte Carlo (MCMC) iterations to obtain the corresponding Ln Pr ( X|K ). The recommendation of Evanno et al. (2005) was used to determine the most probable number of populations. Some values of the number ( K ) of an a priori-defined clusters were compared and were used to calculate the Ln Pr ( X|K ). To validate the structure results, population assignment test was performed using GenAlEx 6.5 (Peakall and Smouse, 2012).

**Table 3:** Intra-population genetic diversity indices estimated using different SSR datasets.

Populations	N	$N_{a}$	$N_{e}$	I	$H_{o}$	$H_{e}$	$F_{\text{IS}}$
*Phenotypes*							
Black	8	3.353	2.517	0.984	0.474	0.558	0.144
Brown	8	3.235	2.254	0.883	0.522	0.503	0.003
Buff	8	3.235	2.374	0.938	0.537	0.540	0.007
Cinnamon	8	3.529	2.609	1.023	0.456	0.578	0.236
Lavender	8	3.412	2.708	1.061	0.471	0.605	0.222
Multicolored	8	3.353	2.404	0.948	0.463	0.534	0.140
Pastel	8	3.059	2.294	0.893	0.471	0.521	0.125
Pearl gray	8	3.353	2.531	0.979	0.500	0.564	0.123
Pied pearl gray	8	3.471	2.591	0.984	0.556	0.549	- 0.010
Pied white	8	3.176	2.520	0.975	0.551	0.564	0.038
Royal purple	8	3.353	2.413	0.933	0.478	0.528	0.140
White	8	2.529	1.827	0.668	0.434	0.412	- 0.083
*Climatic zones*							
Guinean	32	4.235	2.781	1.113	0.490	0.610	0.194
Sudano-Guinean	32	4.059	2.537	1.032	0.474	0.567	0.150
Sudanian	32	4.471	2.782	1.131	0.512	0.608	0.152
Mean	32	4.255	2.700	1.092	0.492	0.595	0.165
SE	0.000	0.190	0.100	0.043	0.025	0.020	0.031

N : number of birds, $N_{a}$ : average number of alleles, $N_{e}$ : effective number of alleles, I : Shannon's information index, $H_{o}$ : observed heterozygosity, $H_{e}$ : expected heterozygosity, $F_{\text{IS}}$ : inbreeding coefficient.

The values for the number of clusters ( K ) were assessed following Evanno et al. (2005), by comparing the estimated posterior probability of data for different values of K and the standard deviation between runs for the same K . The program estimates the posterior distribution ( q ) of each individual's admixture coefficient. The contour maps of the admixture coefficient q were constructed using DISTRUCT software.

## 3 Results

### 3.1 Genetic diversity and polymorphism within guinea fowl population

A total of 83 alleles were identified with an average number of 5.0 ± 0.19 alleles per locus (Table 2). The number of alleles per locus in the whole population varied from two for locus GUJ0084 to eight for locus GUJ0013. The number of effective alleles per locus averaged 2.70 ± 0.10, ranging from 1.137 (GUJ0001) to 3.918 (GUJ0059). The polymorphism information content (PIC) value of the SSR markers, which is a measure of allele diversity at a locus, ranged from 0.626 (GF74) to 0.958 (GUJ0084) with an average value of 0.793 ± 0.108. All (100 %) of the 17 loci studied had a PIC value greater than 0.5 (Table 2). The observed heterozygosity (Ho) ranged from 0.125 (GUJ0001) to 0.781 (GUJ0013), with an average of 0.492 ± 0.025 across all subpopulations. With regards to the expected heterozygosity ( $H_{e}$ ), it varied from 0.463 for locus GUJ0084 to 0.745 for locus GUJ0059, with an average of 0.595 ± 0.020. The majority (70.6 %) of the loci showed a heterozygote deficit ( $H_{o}$ < $H_{e}$ ) according to the Hardy–Weinberg equilibrium, except the loci GF12, GUJ0013, GF74, GUJ0001 and GF75. Shannon's information index ranged from 0.269 (GUJ0001) to 1.299 (GUJ0013) with a mean of 1.092 (Table 2). The overall deficit of heterozygotes in the total population is reflected by $F_{\text{IT}}$ values ranging from - 0.141 for locus GUJ0013 to 0.519 for GF69 with an average value of 0.193.

### 3.2 Genetic relationship among the 12 phenotypes and 3 climatic zones

The lowest average number of alleles (2.529 alleles) has been observed for the white phenotype, whereas the highest (3.529 alleles) is confirmed for the cinnamon phenotype (Table 3). Apart from the brown, pied pearl gray and white phenotypes, all other phenotypes showed a deficit in heterozygotes. The values of inbreeding coefficient ( $F_{\text{IS}}$ ) ranged from 0.150 for the Sudano-Guinean zone to 0.194 for the Guinean zone, with an average of 0.165. Among the three climatic zones investigated, the mean values of observed alleles ( $N_{a}$ ) and effective alleles ( $N_{e}$ ) were 4.255 and 2.700, respectively. The Sudano-Guinean zone recorded the lowest values of $N_{a}$ (4.059) and $N_{e}$ (2.537). Likewise, the highest values of $N_{a}$ (4.471) and $N_{e}$ (2.782) were recorded in the Sudanian zone. The observed heterozygosity in the Sudanian guinea fowl subpopulation (0.512) was higher than the values obtained in the Guinean (0.490) and Sudano-Guinean (0.474) subpopulations (Table 3).

**Table 4:** Observed heterozygosity, expected heterozygosity and deviation from panmixia of each climatic zone in Benin.

Loci	Guinean zone			Sudano-Guinean zone			Sudanian zone		
	$H_{o}$	$H_{e}$	$F_{\text{IS}}$	$H_{o}$	$H_{e}$	$F_{\text{IS}}$	$H_{o}$	$H_{e}$	$F_{\text{IS}}$
GF12	0.656	0.644	- 0.020	0.594	0.558	- 0.064	0.688	0.735	0.064
GUJ0013	0.875	0.615	- 0.423	0.719	0.675	- 0.064	0.750	0.730	- 0.027
GF168	0.750	0.737	- 0.017	0.656	0.705	0.069	0.625	0.680	0.080
GF43	0.219	0.571	0.617	0.594	0.649	0.085	0.344	0.543	0.367
GF69	0.125	0.625	0.800	0.344	0.709	0.515	0.469	0.541	0.133
GUJ0084	0.281	0.390	0.279	0.281	0.500	0.437	0.469	0.500	0.062
GUJ0059	0.438	0.744	0.412	0.531	0.739	0.281	0.594	0.751	0.210
GF13	0.500	0.682	0.266	0.375	0.448	0.162	0.531	0.653	0.186
GF74	0.844	0.727	- 0.161	0.500	0.519	0.036	0.594	0.643	0.077
GF5	0.469	0.615	0.238	0.375	0.539	0.304	0.469	0.614	0.236
GUJ0001	0.188	0.174	- 0.076	0.063	0.061	- 0.024	0.125	0.119	- 0.053
GF30	0.438	0.646	0.323	0.563	0.567	0.009	0.625	0.713	0.124
GF75	0.594	0.635	0.065	0.719	0.555	- 0.296	0.469	0.573	0.182
MCW0069	0.500	0.597	0.163	0.500	0.638	0.217	0.406	0.653	0.378
GF37	0.625	0.700	0.107	0.469	0.719	0.348	0.625	0.676	0.076
GUJ0086	0.531	0.656	0.190	0.375	0.539	0.304	0.531	0.625	0.149
GUJ0066	0.290	0.618	0.530	0.406	0.528	0.231	0.393	0.589	0.333
Mean	0.490	0.610	0.194	0.474	0.567	0.150	0.512	0.608	0.152
Standard error	0.054	0.034	0.073	0.041	0.038	0.051	0.036	0.035	0.030

Ho: observed heterozygosity, He: expected heterozygosity, $F_{\text{IS}}$ : inbreeding coefficient.

### 3.3 Genetic diversity parameters of each climatic zone

The Guinean zone recorded the highest inbreeding index (0.194), while the Sudano-Guinean zone recorded the lowest inbreeding index (0.150). In the Guinean zone, 70.6 % of loci show a deficit of heterozygotes. A deficit of heterozygotes has been also obtained in the Sudano-Guinean and the Sudanian zones, with the proportions of 76.5 % and 88.2 %, respectively (Table 4). Only five, four and two negative values of the inbreeding index have been obtained in the Guinean, Sudano-Guinean and Sudanian zones, respectively. Each of the three subpopulations of climatic zones showed relatively high positive values of $F_{\text{IS}}$ , indicating a deficit of heterozygotes.

**Table 5:** Pairwise population matrix of Nei unbiased genetic identity (below triangular matrix) and genetic distance (above triangular matrix).

Black	Brown	Buff	Cinnamon	Lavender	Multicolored	Pastel	Pearl gray	Pied pearl gray	Pied white	Royal purple	White	
–	0.060	0.080	0.138	0.031	0.029	0.144	0.084	0.031	0.103	0.207	0.198	Black
0.942	–	0.054	0.256	0.039	0.060	0.161	0.166	0.192	0.177	0.187	0.050	Brown
0.923	0.948	–	0.067	0.024	0.031	0.000	0.140	0.135	0.158	0.070	0.077	Buff
0.871	0.774	0.935	–	0.021	0.082	0.014	0.093	0.080	0.182	0.136	**0.347**	Cinnamon
0.970	0.962	0.976	0.979	–	0.005	0.034	0.051	0.081	0.042	0.070	0.118	Lavender
0.971	0.942	0.969	0.921	0.995	–	0.067	0.039	0.001	0.163	0.126	0.156	Multicolored
0.866	0.851	**0.998**	0.986	0.967	0.936	–	0.176	0.161	0.205	0.065	0.190	Pastel
0.919	0.847	0.869	0.911	0.951	0.961	0.839	–	**0.001**	0.233	0.190	0.286	Pearl gray
0.969	0.825	0.874	0.923	0.922	0.990	0.851	0.992	–	0.225	0.216	0.341	Pied pearl gray
0.902	0.838	0.854	0.834	0.959	0.850	0.815	0.792	0.799	–	0.184	0.245	Pied white
0.813	0.830	0.932	0.873	0.933	0.882	0.937	0.827	0.806	0.832	–	0.252	Royal purple
0.820	0.952	0.926	**0.707**	0.889	0.855	0.827	0.751	0.711	0.783	0.778	–	White

NB: extreme values (minimum and maximum) are in bold.

**Figure 3:**
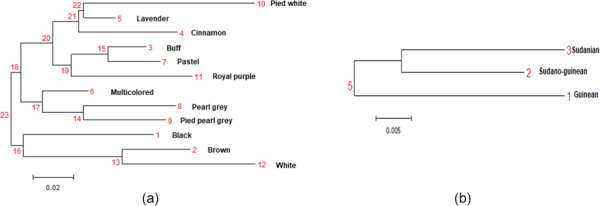
Phylogenetic tree representing Nei's genetic distance between **(a)** the 12 phenotypes of indigenous guinea fowl and **(b)** the climatic zones.

### 3.4 Population relationship and structure

The genetic distances between phenotypes are low, ranging from 0.001 (pied pearl gray and pearl gray) to 0.347 (cinnamon and white), unlike the strong genetic identities (ranging from 0.707 to 0.998) that reflect a strong genetic similarity among the 12 phenotypes of indigenous guinea fowl studied (Table 5). The phylogenetic tree obtained from the pairwise genetic distance of Nei (1978) between subpopulations shows two separated groups, and this clustering is supported by 23 % bootstrap values. Black, brown and white phenotypes formed a branch. In group two, there are two subgroups supported with 18 % bootstrap values. Group 2 subgroup 1 includes multicolored, pearl gray, and pied pearl gray, and all the other six phenotypes formed a separate large group with branches (Fig. 3a). The subpopulations of the Sudano-Guinean and Sudanian zones tended to cluster together, while the subpopulation of the Guinean zone appeared to be relatively distinct from them (Fig. 3b).

**Figure 4:**
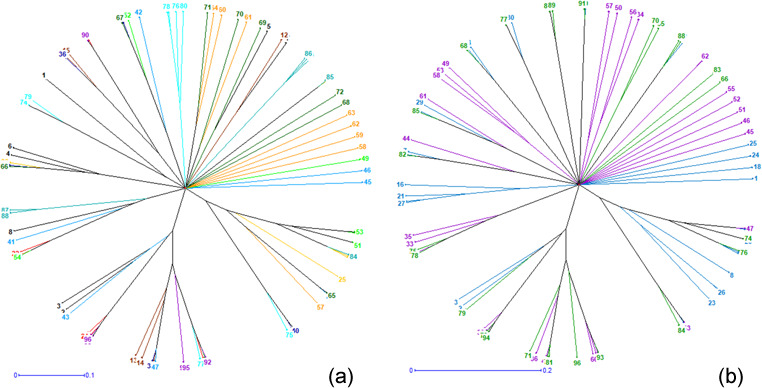
Neighbor-joining tree relating the 96 individuals of **(a)** the 12 phenotypes and of **(b)** the 3 climatic zones.

**Figure 5:**
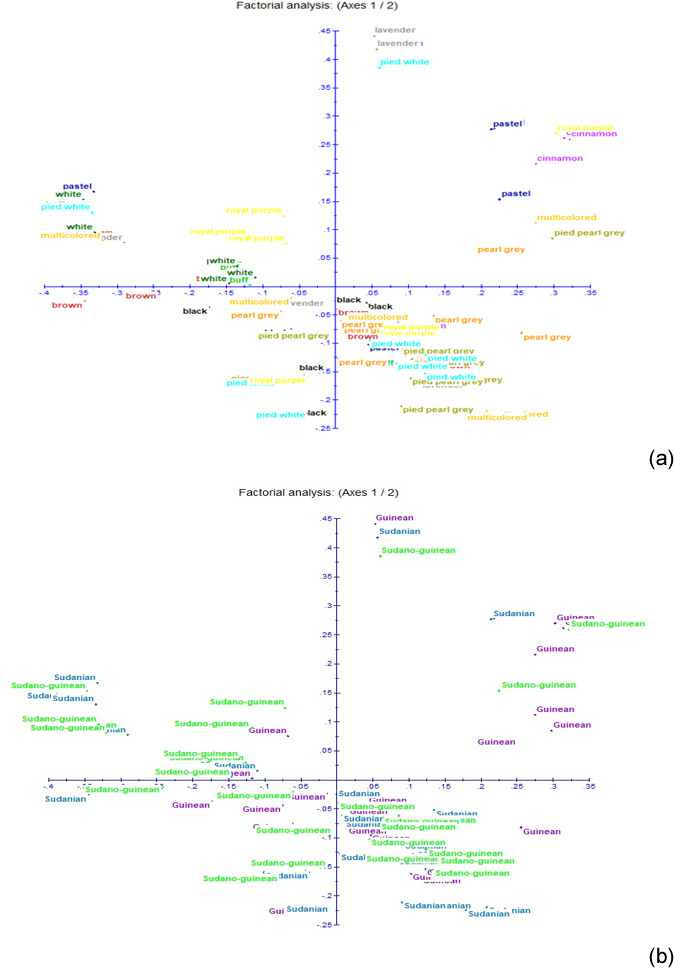
Results of factorial correspondence analysis showing **(a)** the relationship between the phenotypes and **(b)** the relations between guinea fowl subpopulations of the climatic zones.

**Figure 6:**
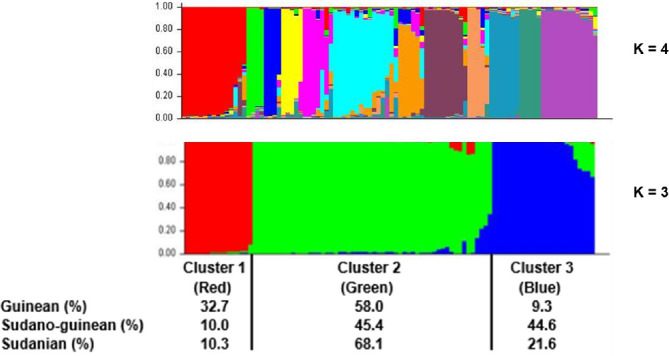
Population structure of the three climatic zones for k=3 and k=4 . In the clustering diagram with k=3 , each line corresponds to an individual, and the shading indicates the three population clusters (cluster 1: red, cluster 2: green, and cluster 3: blue). The values on the x axis represent the proportions of individuals from each climatic zone within the respective clusters.

The neighbor-joining tree derived from pairwise inter-individual allele sharing distances revealed an admixture of individuals from all the 12 phenotypes (Fig. 4a) and from the 3 climatic zones (Fig. 4b). The results of factorial correspondence analysis showed a low degree of genetic diversity between the 12 indigenous guinea fowl phenotypes studied (Fig. 5a) and between the 3 subpopulations of the climatic zones studied (Fig. 5b). Among the three groups obtained at K = 3, which is the optimal number of clusters, Cluster 1 consisted mostly of individuals (32.7 %) from the Guinean zone. Cluster 2 included 68.1 % and 58 % of individuals from the Sudanian and Guinean zones, respectively. Cluster 3 predominated with individuals (44.6 %) from the Sudano-Guinean zone (Fig. 6).

## 4 Discussion

The results showed that all loci were polymorphic with an average polymorphism information content value of 0.793, considered to reflect informative loci (Botstein et al., 1980). Based on the classification suggested by these authors, 100 % ( 17/17 ) of the SSR markers used in this study were highly informative (PIC > 0.50). The panel of 17 SSR markers used in the present study is therefore suitable for a genetic evaluation of the indigenous guinea fowl populations in Benin. A total of 83 alleles were found for the 17 loci studied, with an average number of 5.0 alleles per locus. This is lower than the number reported in guinea fowl populations in other West African countries (11 alleles per locus) by Kayang et al. (2010), who used 6 SSR markers and 190 samples, as well as in wild and domestic guinea fowl in the Republic of the Sudan (9.7 alleles per locus) by Weimann et al. (2016), who used 25 SSR markers to genotype 184 samples. Similarly, Traoré et al. (2018) in Burkina Faso, who used 19 SSR markers developed in guinea fowl, chicken and quail to analyze 190 samples, obtained higher values (7.16 alleles per locus) than those obtained in this study, which characterized 96 samples using 17 microsatellite markers developed in guinea fowl, quail and chicken. On the other hand, the number of alleles obtained in this study is close to the results of Soara et al. (2022) in local populations of guinea fowl in north Togo, where the climatic conditions are similar to those in northern Benin. Indeed, Soara et al. (2022) used 18 SSR markers to genotype 94 samples. Locus GUJ0013 showed the highest number of polymorphisms in our sample (eight alleles). The values obtained are lower than those of Weimann et al. (2016) in the Republic of the Sudan and Traoré et al. (2018) in Burkina Faso with 36 alleles and 16 alleles, respectively, which used approximately double the number of samples used in this study, and the climatic conditions of the countries are not as superimposable. The differences observed between these studies, added to the environmental, biological and historic differences between the target populations, could also be explained by the samples size and by the SSR markers used for genotyping. Indeed, the nature of the loci used influences the average number of alleles per locus, and the number of alleles observed in a given locus in a population tends to increase with the size of the sample examined, which means that this parameter must be taken into account in comparisons of genetic diversity between populations (Ollivier and Foulley, 2013). In the set of loci used in this study, the observed (0.492) and expected (0.595) heterozygosities were high. The white (albino) phenotype showed the lowest heterozygosity ( $H_{o}$ = 0.434 and $H_{e}$ = 0.412) values. These findings were similar to the results found by Soara et al. (2022) in guinea fowl populations of northern Togo. The higher overall rates of heterozygosity indicate a high genetic diversity, which is consistent with the great phenotypic variability observed previously in the same subpopulations (Orounladji et al., 2021). However, observed heterozygosity lower than that expected under the Hardy–Weinberg equilibrium hypothesis reflects a deficit of heterozygotes in the population that could be related to inbreeding or technical problems of amplification generating the null alleles. A deficit of heterozygotes has been obtained in all the subpopulations studied as also reflected by the positive value of the inbreeding index. Wright's fixation indices ( $F_{\text{IS}}$ = 0.167, $F_{\text{ST}}$ = 0.033 and $F_{\text{IT}}$ = 0.193) found in indigenous guinea fowl population of Benin were relatively low. Similar results were reported in indigenous guinea fowl from the Republic of the Sudan, Burkina Faso and Togo (Weimann et al., 2016; Traoré et al., 2018, Soara et al., 2022). The low $F_{\text{ST}}$ value (0.033) means that only 3.3 % of the total genetic diversity could be assigned to differences between subpopulations, suggesting a low substructuring of the studied population like in northern Togo (Soara et al., 2022). The inbreeding coefficient of 16.7 % suggests a significant degree of inbreeding and is a consequence of mating of related individuals. However, the increase in $F_{\text{IS}}$ cannot be systematically linked to inbreeding as the presence of null alleles could contribute to the heterozygote deficit (Kelly et al., 2011; Huang et al., 2016). This heterozygote deficit may be due to the occasional failure of amplification leading to the presence of alleles. This phenomenon is generally a common difficulty for SSR marker analysis (Ollivier, 2009). The genetic distances between phenotypes were low, unlike the strong genetic identities that reflect a strong genetic similarity between the 12 phenotypes of indigenous guinea fowl studied. These results indicated the existence of a single indigenous guinea fowl population with a high intra-population genetic diversity (phenotypic or climatic zone).

The low difference in the genetic variability between the studied phenotypes was consolidated by the high values of genetic identity and the low genetic distances between phenotypes and between climatic zones. Some authors (Kayang et al., 2010; Weimann et al., 2016; Soara et al., 2022) also reported low values of genetic distances. A genetic diversity study in Togo showed a very high and almost identical genetic similarity (0.98–0.99) between pearl gray, Bonaparte and multicolored guinea fowl phenotypes (Soara et al., 2022). In our study, only brown, white and pied white phenotypes, over the 12 phenotypes, stood out slightly from the others. These findings are evidence of a weak genetic variation between the phenotypes (based on the feathers, plumage and color) and between the three climatic zones. The observed low genetic differentiation could be ascribed to the many years of non-selective breeding (based on feather color) and to the uncontrolled movements of birds between climatic zones and between border countries, which promotes gene flow (Weimann et al., 2016; Traoré et al., 2018 ; Soara et al., 2022). In addition, the SSR markers used in the present study are neutral markers and, therefore, are not involved in the determinism of the expression of feather coloring genes. The three climatic guinea fowl subpopulations are also genetically close (genetic identity ranging from 0.904 to 0.941). It would be interesting to include in future studies wild subpopulations from the forests of Benin as well as guinea fowl populations from neighboring countries such as Togo, Burkina Faso, Niger and Nigeria. These findings could serve as baseline information for breeding strategies for the conservation and improvement of indigenous guinea fowl populations in Benin. It is necessary to improve the farming conditions such as the control of habitat, diet and diseases as suggested by our previous study (Orounladji et al., 2022). The 12 guinea fowl phenotypes identified in our studies on morphobiometric characterization and biodiversity of guinea fowl (Orounladji et al., 2021) are found to be from a single population.

## 5 Conclusions

The molecular genetic characterization that was carried out during the present study made it possible to determine the genetic diversity and structuring of indigenous guinea fowl populations in Benin. The study showed that all the microsatellite markers used are polymorphic and have a strong informative capacity on the genetic diversity within the indigenous guinea fowl population studied. A deficit of heterozygotes was observed in the overall population and in the subpopulations represented by the phenotypes, as well as weak genetic structuring in the local guinea fowl of Benin. Due to the weak differentiation between guinea fowl populations in the three climatic zones, the future breeding strategy or program should not take into account the different phenotypes. A breeding program could be developed and implemented for better management of the diversity existing within and between recorded guinea fowl subpopulations and for the sustainable production of this poultry species in Benin. Any program or strategy needs to take into account not only the different uses of the species (income generation, consumption social, cultural and cultural roles, etc.) but also the local community preferences. An ecological niche modeling could be also carried out to understand to what extent they are tolerant to environmental factors and to predict their dynamics until 2085 based on the recommendations of the Intergovernmental Panel on Climate Change.

## Data Availability

The data presented in this study are available upon request from the corresponding author.

## Sample availability

Samples were taken with the consent of the farmer and related personnel.
